# Thyroid Dysfunction and Sleep Disorders

**DOI:** 10.3389/fendo.2021.725829

**Published:** 2021-08-24

**Authors:** Max E. Green, Victor Bernet, Joseph Cheung

**Affiliations:** ^1^Mayo Clinic School of Graduate Medical Education, Mayo Clinic College of Medicine and Science, Jacksonville, FL, United States; ^2^Division of Endocrinology, Mayo Clinic, Jacksonville, FL, United States; ^3^Department of Neurologic Surgery, Mayo Clinic, Jacksonville, FL, United States; ^4^Division of Pulmonary, Allergy and Sleep Medicine, Mayo Clinic, Jacksonville, FL, United States

**Keywords:** hyperthyroidism, hypothyroidism, insomnia, restless legs syndrome, sleep, sleep apnea, obstructive, sleep initiation and maintenance disorders

## Abstract

Thyroid disorders and sleep disorders are common problems in the general population that can affect people of all ages, backgrounds, and sexes, but little is known about their clinical associations. We reviewed the literature assessing the associations between thyroid disease and sleep disorders and noted that hyperthyroidism and hypothyroidism have clinical overlap with sleep conditions such as insomnia, restless legs syndrome, and obstructive sleep apnea. These findings highlight the importance of identifying and managing thyroid dysfunction for patients with these common sleep disorders. Additional research is needed to further understand how thyroid dysfunction affects sleep physiology.

## Introduction

Millions of Americans have sleep-related disturbances each year, and extensive research has shown that inadequate or dysfunctional sleep has a far-reaching health impact. Although the cause, severity, and consequences of sleep disturbances vary widely across the spectrum of individuals, a large portion of sleep medicine research focuses on the impact of sleep disorders (e.g., insomnia, obstructive sleep apnea [OSA]) on cardiovascular and neurologic health. Consequently, the effects of sleep disorders on many other organ systems have been explored in considerably less detail. The purpose of this paper is to discuss the effects of thyroid function on sleep and highlight the relationships between thyroid pathologies and sleep health. Additionally, we explore the impact of common sleep disorders on the health and function of the thyroid gland.

## Thyroid Dysfunction

The thyroid produces 2 main hormones, thyroxine (T4) and triiodothyronine (T3), which affect numerous physiologic processes in the body, including body temperature maintenance, digestion, and vital functions such as heart rate and respiration (1). Symptoms that develop because of improper production of these hormones can have far-reaching effects on the body and may vary immensely in severity, depending on the cause of the dysfunction. Hypothyroidism, which generally is caused by an underactive thyroid, affects nearly 5% of the US population. Hyperthyroidism, which generally is caused by an overactive thyroid, affects approximately 1% of the US population (2). Both conditions usually are treated pharmacologically, either by replacing the thyroid hormone that is missing or by trying to block the production or effect (or both) of the excess thyroid hormone.

Although thyroid dysfunction is known to affect many bodily systems, the relationship between thyroid disorders and sleep function currently is not well understood. Because sleep disorders are rarely the sole presenting symptom of thyroid dysfunction, it is important to consider the relationship between thyroid function and sleep when providing a whole-body treatment approach for patients with these disorders.

## Insomnia

### Hyperthyroidism

Hyperthyroidism, defined as the presence of increased thyroid activity, and thyrotoxicosis, defined as the presence of too much thyroid hormone, are common and well-known causes of sleep dysfunction. Often, sleep disturbances associated with hyperthyroidism are caused by hyperkinetic features of the disorder. Stern et al. (3) assessed 137 patients with Graves disease, the most common cause of hyperthyroidism, and reported that 66.4% of study participants had difficulty falling asleep. Sridhar et al. (4) suggested that elevated levels of thyroid hormone were associated with several components of sleep dysfunction, including prolonged sleep latency, difficulty maintaining sleep, and excessive daytime sleepiness. Specifically, thyroid hormone−mediated changes in appetite, bowel movements, and mood (eg, increased anxiety) were associated with significantly prolonged sleep latency. Similarly, patients with tremor caused by elevated thyroid hormones had markedly increased difficulty in maintaining sleep. Xia et al. (5) showed a direct correlation between levels of thyroid-stimulating hormone (TSH), T3, and T4 and the severity of insomnia symptoms.

Additionally, hyperthyroidism can cause or worsen other conditions such as anxiety or depression, which in turn can further impair sleep and insomnia (6). Chattopadhyay et al. (7) assessed a group of 36 patients in India with newly diagnosed Graves disease and comorbid psychiatric concerns, including 41% with generalized anxiety disorder, 16% with obsessive-compulsive disorder, and 16% with undifferentiated mood disorders. The patients reported primary concerns about insomnia, irritability, and anxiety. The study divided patients into 2 treatment groups. One group was treated with antithyroid and antipsychotic medications, and the other group was treated with only antithyroid drugs. The study showed significant improvement in symptoms of insomnia, irritability, and anxiety for both groups. However, the extent of symptomatic improvement was not significantly different between groups. These findings support the idea that disordered sleep and psychiatric problems are closely tied to excessive thyroid activity, and treatment of the thyroid dysfunction can improve or resolve the associated psychiatric symptoms.

### Hypothyroidism

Hypothyroidism, defined as the decreased production of thyroid hormones, may affect overall sleep quality. Although no direct biochemical connection has been established between hypothyroidism and insomnia, some studies have shown a relationship between untreated subclinical hypothyroidism and poor sleep quality. Song et al. (8) showed that people with lower thyroid hormone levels or even subclinical hypothyroidism generally have longer sleep latency, shorter sleep duration, and lower satisfaction with their sleep quality compared with euthyroid individuals. However, a study by Akatsu et al. (9) did not show a relationship between subclinical hypothyroidism and sleep quality. Both studies had relatively small sample sizes, and neither study effectively investigated or identified the actual mechanisms by which lower thyroid hormone levels might affect sleep quality.

One possible reason why hypothyroidism and insomnia may commonly co-occur is because the symptoms associated with thyroid hormone deficiency may contribute to insomnia. For example, underactive thyroid is associated with muscle and joint pain, cold intolerance, and increased anxiety, and these symptoms can contribute to sleep deficiencies. Budhiraja et al. (10) suggest that a higher number of medical comorbidities is associated with a higher risk of insomnia. Even if thyroid hormone deficiency is not directly causing insomnia, the wide range of symptoms associated with thyroid dysfunction can easily exacerbate sleeping difficulties and reduce a person’s ability to achieve quality, restful sleep.

## Obstructive Sleep Apnea

OSA is another common sleep disorder with many causes, and it affects a large portion of the general population. One of the most rigorous population-based studies of OSA was conducted by Young et al. (11), who reported that among adults aged 30 to 60 years, the prevalence of OSA was 9% for women and 24% for men. Although thyroid dysfunction is generally not thought to be among the primary causes of OSA, studies have shown that they appear to be related. Thavaraputta et al. (12) used a multivariate logistic regression analysis to show a significant association between hypothyroidism and OSA after adjusting for demographic characteristics, health care access, body mass index, alcohol use, smoking, and other comorbidities that are common among patients with OSA and hypothyroidism. Resta et al. (13) evaluated obese patients with and without sleep disturbances and showed a higher prevalence of hypothyroidism among patients referred to a sleep clinic for sleep-disordered breathing.

The aforementioned studies support the hypothesis that hypothyroidism may contribute to OSA. However, the specific pathophysiologic mechanism remains relatively elusive. Mete et al. (14) described several mechanisms by which hypothyroidism may be associated with OSA symptoms. For example, some patients with hypothyroidism may have increased thyroid size that causes or worsens upper airway obstruction. Hypothyroidism also could alter ventilatory drive and respiratory muscle function.

The effects of hypothyroidism treatment on OSA have been mixed. Lin et al. (15) showed that T4 replacement therapy improved symptoms of OSA for some patients. This finding was corroborated by Kittle and Chaudhary (16), who showed that thyroid hormone therapy could diminish (or in some cases, completely eliminate) apneic episodes and arterial oxygen desaturations, thus improving sleep satisfaction and sleep efficiency. However, both studies reported that some patients did not have improvements in their sleep experience with thyroid hormone replacement therapy. Bielicki et al. (17) indicated that although hypothyroidism may be a contributing, if not causal, component of OSA for some patients, most patients with OSA have normal thyroid function or do not have improvement in sleep symptoms with thyroid treatment. In fact, many studies, including one by Bruyneel et al. (18), showed that TSH and T4 levels were not significantly different among patients with moderate or severe OSA, suggesting that thyroid hormone levels are not necessarily a marker of OSA severity. Similarly, Mete et al. (14) assessed 150 patients with polysomnographically diagnosed OSA (50 mild, 50 moderate, and 50 severe OSA cases) and showed no significant association between OSA severity and thyroid hormone levels.

Interestingly, Petrone et al. (19) evaluated 125 patients with moderate to severe OSA plus 60 control patients with normal nocturnal respirations and showed that 10.4% of patients with OSA had nonthyroidal illness syndrome (NTIS), defined as normal TSH and low T3 levels. Another 8% in the OSA group had subclinical hypothyroidism, defined as elevated TSH and normal T4 levels. No patients in the control group had NTIS or subclinical hypothyroidism. Patients with NTIS had decreased mean nocturnal oxygen saturations and increased time with oxygen saturation below 90%. After treatment with continuous positive airway pressure, 100% of patients with NTIS had T3 levels increase to the normal range, and 75% of patients with subclinical hypothyroidism had a decrease in TSH levels. No hormonal changes were seen with any of the control patients, who had normal levels.

A study by Bahammam et al. (20) evaluated 271 patients referred for sleep studies and measured the patients’ TSH and T4 levels to determine the prevalence of thyroid disease. Among the patients with confirmed OSA, the prevalence of newly diagnosed clinical hypothyroidism was 0.4%, whereas it was 1.4% in patients without OSA. However, the prevalence of newly diagnosed subclinical hypothyroidism was 11.1% for patients with OSA and 4% of patients without OSA.

These studies illuminate the relationship between hypothyroidism and OSA but also highlight the heterogeneity of causes and contributing factors that can influence a patient’s symptoms. Although many studies support the hypothesis that OSA is associated with thyroid dysfunction, the level of thyroid dysfunction does not appear to predict OSA severity and the severity of OSA may have subtle effects on thyroid hormone levels. Currently, the exact mechanism underlying the interplay between OSA and thyroid function remains unclear.

## Restless Legs Syndrome

A well-known example of thyroid dysfunction contributing to sleep disturbances is abnormal thyroid function that increases the risk of restless legs syndrome (RLS). People with RLS have an uncomfortable or unpleasant sensation in their legs or body when they rest. Thus, RLS symptoms, which usually occur when a person is trying to sleep, can lead to insomnia and sleep dysfunction (21). Conditions with higher levels of thyroid hormones (eg, pregnancy, Graves disease) also are associated with a higher prevalence of RLS symptoms.

Although the exact pathophysiology of RLS is still being investigated, Pereira and Andersen (22) hypothesized that the dopaminergic system has an important role, given the effectiveness of dopamine agonists in alleviating RLS symptoms. Tan et al. (21) suggested that elevated levels of thyroid hormone can be an inciting stimulus for RLS-like symptoms, such as tremors, hyperkinetic states, and insomnia. Their study evaluated 146 patients with biochemically confirmed thyroid disorders and 434 control patients without thyroid disorders. Twelve patients (8.2%) with confirmed thyroid disorders had RLS-like symptoms, whereas only 4 control patients (0.9%) were similarly affected. Furthermore, of the 12 patients with thyroid disorders and RLS-like symptoms, 4 (33%) had complete resolution of RLS symptoms with adequate thyroid treatment. These findings underscore the idea that hyperthyroidism and hypothyroidism may exacerbate RLS symptoms, even if they are not the primary drivers of the disorder. Notably, they showed no significant difference in the prevalence of RLS between patients with and without thyroid dysfunction.

Ahmed et al. (23) reported that RLS symptoms were considerably more prevalent in patients with hypothyroidism than in patients with normal thyroid function. Additionally, patients who had hyperthyroidism before their hypothyroidism, which can commonly occur in autoimmune processes such as Hashimoto thyroiditis, were considerably more likely to have RLS symptoms compared with patients who did not have hyperthyroidism before hypothyroidism. Pradella-Hallinan et al. (24) showed that patients with RLS and subsequent Graves disease had worsening of their RLS symptoms during the hyperthyroid state. Several patients in that study had new-onset RLS after Graves disease developed.

Although thyroid dysfunction does not appear to directly cause RLS, it does affect RLS symptoms. With this in mind, thyroid hormone levels are a potentially modifiable risk factor for RLS, and clinicians should consider correcting thyroid abnormalities to minimize the symptoms of RLS and their effects on sleep.

## Conclusion

Thyroid dysfunction can contribute to a myriad of symptoms that involve nearly every system in the body, including sleep function. Even though current evidence suggests that thyroid hormone levels are not markers of sleep dysfunction, untreated thyroid dysfunction clearly can affect a person’s ability to achieve healthy, restful sleep. Although sleep dysfunction is not among the most common symptoms that clinicians associate with thyroid disorders, thyroid and sleep dysfunction commonly co-occur. Clinicians should keep this association in mind when using a whole-body approach to treat patients with thyroid dysfunction and sleep disorders.

## Author Contributions

MG: Data collection, literature review, manuscript writing and revision. VB: Consultation and expert advice, manuscript revision, and content recommendation. JC: Primary research advisor, data collection, literature review, manuscript writing and revision. All authors contributed to the article and approved the submitted version.

## Funding

JC is supported by the Mayo Clinic in Florida Research Accelerator for Clinicians Engaged in Research Program and the Department of Medicine Catalyst for Advancing in Academics award.

## Conflict of Interest

The authors declare that the research was conducted in the absence of any commercial or financial relationships that could be construed as a potential conflict of interest.

## Publisher’s Note

All claims expressed in this article are solely those of the authors and do not necessarily represent those of their affiliated organizations, or those of the publisher, the editors and the reviewers. Any product that may be evaluated in this article, or claim that may be made by its manufacturer, is not guaranteed or endorsed by the publisher.

## Abbreviations

NTIS, nonthyroidal illness syndrome; OSA, obstructive sleep apnea; RLS, restless legs syndrome; T3, triiodothyronine; T4, thyroxine; TSH, thyroid-stimulating hormone.
